# The Roles of Sirt1 in Breast and Gynecologic Malignancies

**DOI:** 10.3390/biology14111510

**Published:** 2025-10-28

**Authors:** Jianmin Ding, Matthew T. Ye, Songlin Zhang

**Affiliations:** 1 Department of Pathology & Immunology, Baylor College of Medicine, Houston, TX 77030, USA; jianmin.ding@bcm.edu (J.D.); matthewye88@gmail.com (M.T.Y.); 2 School of Medicine, Vanderbilt University, Nashville, TN 37204, USA

**Keywords:** SIRT1, breast cancer, gynecologic malignancies, SIRT1 inhibitors, biomarker

## Abstract

Sirtuin 1 (SIRT1), an NAD^+^-dependent histone deacetylase, exerts complex and context-dependent effects in breast and gynecological cancers. By deacetylating histone and non-histone proteins such as p53, FOXO, and NF-κB, SIRT1 regulates essential processes including DNA repair, apoptosis, metabolism, and stress response. In breast cancer, SIRT1 may act as a tumor suppressor in early stages by maintaining genomic stability but promotes epithelial–mesenchymal transition, metastasis, and chemoresistance in aggressive subtypes such as triple-negative breast cancer. Similarly, in gynecological cancers, SIRT1 displays dual roles: promoting proliferation via estrogen signaling and p53/FOXO1 inhibition in Type I endometrial cancer yet potentially supporting DNA repair in high-grade Type II tumors. Its overexpression in ovarian and cervical cancers is linked to enhanced survival and drug resistance. Preclinical studies show that pharmacological inhibition of SIRT1 (e.g., with EX-527 or cambinol) restores chemosensitivity and reduces tumor cell viability, suggesting potential for SIRT1 inhibitors as adjuncts in cancer therapy. However, clinical trials specifically targeting SIRT1 in these cancers remain limited. Further investigation is needed to define therapeutic windows, molecular contexts, and combination strategies that could optimize SIRT1-targeted therapies. This review summarizes the current understanding of SIRT1’s roles in breast and gynecologic malignancies.

## Simple Summary

Breast and gynecological cancers remain major health challenges, especially when they become resistant to standard treatments. One protein that may play a key role in these cancers is Sirtuin 1 (SIRT1), which helps control important cell functions like DNA repair, cell survival, and stress response. Depending on the cancer type and stage, SIRT1 can either slow down cancer growth or help it spread and resist treatment. In early-stage breast cancer, SIRT1 may protect cells by keeping their DNA stable. But in more aggressive forms, such as triple-negative breast cancer, it can support tumor growth, spread, and resistance to chemotherapy. A similar pattern is seen in gynecological cancers like endometrial, ovarian, and cervical cancer. In lab studies, blocking SIRT1 with experimental drugs has been shown to make cancer cells more sensitive to chemotherapy and reduce their ability to survive. However, clinical trials in patients are still very limited. This review highlights what scientists currently know about SIRT1 in these cancers and suggests that, with further research, targeting SIRT1 could help improve outcomes for patients, especially those with hard-to-treat tumors. Understanding how and when to block SIRT1 could lead to more effective, personalized cancer therapies in the future.

## 1. Introduction

Sirtuin 1 (SIRT1) is a protein that acts as a nicotinamide-adenine-dinucleotide (NAD^+^)-dependent enzyme. It works as both a deacetylase and an ADP-ribosyltransferase. SIRT1 removes acetyl groups from proteins and transfers ADP-ribose groups to them using NAD^+^ as a coenzyme. SIRT1 belongs to the sirtuin family (class III histone deacetylases), and its structure enables it to perform NAD^+^-dependent deacetylation of various substrates, such as histones and transcription factors. It comprises 747 amino acid residues and is one of seven proteins in the sirtuin family. Through these enzymatic activities, SIRT1 regulates numerous cellular processes, including aging, metabolism, stress responses, and cancer [1,2].

SIRT1 is composed of three functional domains. The N-terminal region contains the nuclear localization signal (NLS), which aids the protein in entering the nucleus. The central core region includes the catalytic domain, which exhibits sirtuin-specific deacetylase activity essential for the protein’s enzymatic function. Finally, the C-terminal region features a regulatory domain that influences the protein’s activity and its interactions with other molecules [3].

SIRT1 modulates the acetylation of histones and non-histone proteins (e.g., p53, FOXO, NF-κB), influencing gene expression, DNA repair, apoptosis, and metabolism. In breast and gynecologic cancers, SIRT1 exhibits context-dependent roles, functioning as either a tumor suppressor or tumor promoter depending on the cellular environment and cancer subtype.

## 2. SIRT1 Structure and Functions

Eukaryotic nuclear DNA wraps around a positively charged core of histone proteins. Two copies of histone H2A, H2B, H3, and H4 proteins constitute this protein core. The differential post-translational modifications of the N-terminal tails of these histone proteins have great consequences for gene expression. Acetylation of lysine residues by histone acetyltransferases (HATs) at the ε-ammonium group generally promotes gene expression, resulting in looser, more accessible chromatin. In contrast, deacetylation of these residues through histone deacetylases (HDACs) typically silences gene expression. The reversibility of these modifications results in a dynamic nature to gene expression, constituting a “histone code”.

The 18 human HDACs fall under four classes through their homology to yeast HDACs. The class I Rpd3-like proteins utilize a zinc-ion catalyst for the hydrolysis of acetyl groups, releasing free acetate into the environment. The class II Hda1-like proteins follow a similar zinc-dependent mechanism. The class IV HDAC protein shares much sequence homology with class I and II proteins [4]. By contrast, Class III Sir2-like proteins, known as sirtuins, use NAD^+^ as a helper molecule (cofactor) to remove acetyl groups from proteins. During this process, the acetyl group is transferred to ADP-ribose, producing a molecule called nicotinamide. Nicotinamide can then act as a feedback inhibitor, slowing down the activity of sirtuins [5].

The N-terminal domain of SIRT1 plays important regulatory roles. It facilitates protein–protein interactions, allowing interactions with transcription factors and co-regulators such as p53, E2F1, FOXO transcription factors, and DBC1, an endogenous inhibitor of SIRT1 [6]. The N-terminal domain contains two nuclear localization signals (NLSs) and two nuclear export signals (NESs), enabling SIRT1 to shuttle between the nucleus and cytoplasm [7]. Such dynamic localization is vital for SIRT1’s role in various cellular processes and is influenced by factors like oxidative stress and signaling pathways [6,8]. The N-terminal region also mediates interactions with several regulatory proteins, including AROS (Active Regulator of SIRT1), which can modulate SIRT1’s activity. These interactions are essential for the fine-tuning of SIRT1’s functions in response to cellular cues [6,7,9]. Moreover, this domain is subject to various post-translational modifications, such as phosphorylation. For instance, phosphorylation at specific serine and threonine residues can enhance SIRT1’s nuclear localization and enzymatic activity, particularly under stress conditions [8,10].

The catalytic domain of the Sir2 family members is highly conserved. A zinc ribbon stabilizes the NAD+ domain, which is arranged in a major Rossman fold that binds the nucleotide. The deacetylation reaction occurs at a cleft between these two substructures [11]. The catalytic domain of human SIRT is flanked by N- and C-terminal domains, which are variable and subject to post-translational modifications [8]. Interestingly, unlike those of other Sirtuins, the catalytic domain of SIRT1 has been demonstrated to be inactive in the absence of these terminal domains. A 25-amino acid stretch in the C-terminal domain (known as ESA, or “essential for Sirtuin activity”) interacts with the deacetylase core, greatly potentiating activity. The SIRT1 inhibitor DBC1 competes with ESA to abolish this effect in trans [12].

## 3. SIRT1 Deacetylates Histone and Non-Histone Proteins

### 3.1. SIRT1 Regulates Histone Proteins

SIRT1 is known to deacetylate histone proteins, playing a crucial role in regulating gene expression and chromatin dynamics. By removing acetyl groups from histones, SIRT1 influences the structure of chromatin, leading to changes in transcriptional activity. This deacetylation process typically results in a more compact chromatin structure, which can repress gene expression.

SIRT1 has been observed to deacetylate a variety of histone substrates, which generally results in gene repression, chromatin compaction, and transcriptional silencing. This primarily includes histone 4 at lysine 16; histone 3 at lysine 9, 14, and 56; histone 2AX; and histone 1 at lysine 26. Table 1 provides an overview of SIRT1’s histone targets. Through this activity, SIRT1 can modulate the expression of different downstream targets.

#### 3.1.1. SIRT1 and Acetylated H4K16

Acetylation of H4 at lysine 16 (H4K16Ac) plays a crucial role in chromatin organization [13], being directly responsible for a reduction in inter-nucleosome interactions that are facilitated by the tail of H4. This results in a lack of chromatin compaction and thus increased accessibility to local genetic elements. In yeast, this silencing typically occurs at mating type loci, telomeres, and nucleolar rDNA repeats [14]. In humans, H4K16Ac is also instrumental in the repair of double-stranded breaks, being responsible for recruiting 53BP1 and thus peaking in incidence in S phase [15]. Interestingly, H4K16Ac has been observed to be decreased in aged cells, resulting in impaired repair [16].

While SIRT1 has been demonstrated to deacetylate acetylated H1, H3, and H4 in vitro, previous work has found that it preferentially targets acetylated H4K16 [17]. Furthermore, knockdown of SIRT1 by RNA interference in osteosarcoma resulted in increased levels of hyperacetylation of H4K16 [17]. Meanwhile, resveratrol, a broad sirtuin activator, results in hypoacetylation of H4K16 in *Toxoplasma gondii* [18]. Furthermore, SIRT1 recruitment to a reporter gene located within a region of euchromatin resulted in condensation to heterochromatin [14].

#### 3.1.2. SIRT1 and Acetylated H3K9

Acetylation of histone 3 at lysine 9 (H3K9Ac) is associated with activation of gene expression [19]. H3K9Ac generally plays roles in cell differentiation. It is instrumental in the differentiation of neural stem cells to neurons, with past reports finding H3K9 hypoacetylation at the promoters of oncogenes and cell cycle genes and hyperacetylation in neuron-specific genes in glioblastoma [20]. In zygotes, SIRT1 accumulates in the pronuclei, resulting in the deacetylation of H4K16 and H3K9 [21]. It is believed that the deacetylation of H3K9 exposes the lysine to methylation by histone methyltransferases, an event that is crucial for regulating embryonic development. Indeed, the introduction of BML-278 (a SIRT1-specific activator) to zygotes results in enhanced H3K9 methylation and improved blastocyst formation rates, while the use of an inhibitor (i.e., Sirtinol) results in the opposite effects. Interestingly, the activation of sirtuins with a broad sirtuin activator like resveratrol did not affect blastocyst formation rates, despite inducing hypermethylation of H3K9. This suggests that SIRT1 plays a specific and critical role in embryonic development. Furthermore, SIRT1-mediated hypoacetylation of H3K9 is associated with drug-resistant colony formation and the downregulation of tumor suppressors like SRFP1 and E-cadherin, resulting in the loss of an epithelial phenotype [22]. The interaction between H3K9 and SIRT1 also influences adipocyte development, with SIRT1 activity being associated with reduced adipocyte cytokinesis and proliferation via decreased expression of TRIM1 [23].

SIRT1-mediated deacetylation of H3K9 has also been reported to play roles in reducing or modulating inflammation. For instance, SIRT1 overexpression was shown to prevent the progression of pulmonary fibrosis and aging through hypoacetylation of H3K9 in the promoter of fibroblast-specific *IL-11* [24]. SIRT1 also deacetylates H3K9 in the promoter of *TLR4* in macrophages, thereby preventing activation [25]. This is compounded by interactions with non-histone targets such as NLRP3 and ASC, which are regulators of the macrophage inflammasome. SIRT1 activity in this environment is itself modulated by other enzymes: for instance, PHGDH, an enzyme involved in serine synthesis, consumes NAD+ to decrease the available supply for SIRT1.

#### 3.1.3. SIRT1 and Acetylated H3K14

Like H3K9Ac, acetylation of histone 3 at lysine 14 (H3K14Ac) typically plays roles in activating the expression of products related to development. In fact, past studies suggest that H3K9Ac and H3K14Ac acetylation often regulate the same genes [20]. In addition to development, H3K14Ac is also involved in the hypoxia response. After cells are exposed to MPP, a neurotoxin, SIRT1 is downregulated, resulting in increased acetylation of H3K14 in the promoter HIF-1α [26]. By extension, under typical aerobic conditions, SIRT1 deacetylates H3K14 to repress the expression of HIF-1α. Interestingly, MPP’s role as a neurotoxin mimics the symptoms of Parkinson’s disease, suggesting a link between SIRT1-mediated H3K14 deacetylation and this neurodegenerative disease. Indeed, it was shown that the histone acetyltransferase GCN5 acts antagonistically to SIRT1, increasing H3K14 acetylation, and its loss of function results in a Huntingtin’s disease phenotype in Drosophila [27]. Furthermore, mutation of H3K14 to mimic acetylation (by mutating K to Q) alleviates these cells of the Huntingtin’s disease phenotype.

#### 3.1.4. SIRT1 and Acetylated H3K56

Acetylation of histone 3 at lysine 56 (H3K56Ac) occurs primarily during S-phase and is deacetylated in later stages of the cell cycle [28]. When present, H3K56Ac plays important roles in DNA repair and chromatin assembly after DNA synthesis [29]. The histone chaperones Asf1a and CAF-1 are required for acetylation of H3K56 and the following incorporation of the acetylated histone into chromatin, respectively. The CBP/p300 acetylase is responsible for acetylation. Meanwhile, SIRT1 and SIRT2 are responsible for deacetylation. Increased H3K56Ac, resulting from hyperactive acetylation or improper SIRT1-mediated deacetylation, is associated with high tumor grade and dedifferentiation (though not proliferation). A randomized controlled trial in which patients were administered resveratrol to activate SIRT1 similarly found a reduction in H3K56Ac compared to a placebo [30].

In addition to DNA replication and repair, H3K56Ac also plays a role in transcription. Past work has found that SIRT1 is recruited to sites of H3K56Ac by Rel-A, a subunit of the NF-κB transcription factor complex, at the *BCLAF1* promoter [31]. There, H3K56Ac is deacetylated, resulting in decreased transcription of *BCLAF1*. SIRT1’s deacetylase activity also targets Rel-A itself. Rel-A, which is accelerated by CBP/p300 to activate *BCLAF1* transcription, loses its transcriptional activity when deacetylated. This combined effect to repress *BCLAF1* reduces T-cell proliferation and interleukin-2 production upon TCR stimulation.

#### 3.1.5. SIRT1 and Acetylated H2AX

Histone H2AX is a variant of the core histone H2A that is phosphorylated at Ser 139 by ATM at sites of DNA damage, namely double-stranded breaks (DSBs) [32]. The phosphorylated H2AX proteins form focused clusters that help initiate chromatin remodeling. Cardiotoxicity associated with the chemotherapeutic doxorubicin is believed to stem from dysfunction in this response [33]. Mice with conditional knockout of cardiomyocyte-specific SIRT1 showed increased fibrosis and cardiac dysfunction, as well as increased DNA damage through loss of phospho-H2AX. Critically, there was no mitochondrial dysfunction, which is often associated with the toxic effects of doxorubicin, indicating that SIRT1 has a nuclear-specific protective role. It was concluded that SIRT1 deacetylation of Lys5 was critical for the proper phosphorylation of H2AX Ser139. This pathway was found to be independent of p53, another known modulator of DNA damage repair in cardiotoxicity. Therefore, activation of SIRT1 through resveratrol may protect the heart during doxorubicin administration. A separate investigation suggested an additional indirect pathway of SIRT1-mediated deacetylation of H2AX whereby SIRT1 interacts with and deacetylates Tip60, an acetylase of H2AX Lys 5 [34]. This deacetylation results in the proteasomal-dependent degradation of Tip60, thereby inhibiting the acetylation of Lys 5. Loss of SIRT1-mediated deacetylation of H2AX is also believed to be an indicator of cellular aging [35]. Indeed, medium-dose of formoterol, a β2-adrenergic agonist and activator of SIRT1, protected against senescence in vascular smooth muscle cells, with increased levels of phospho-H2AX being observed [36]. Meanwhile, erythropoietin, which deactivated SIRT1, induced abdominal aortic aneurysm in mice, with decreased phosphor-H2AX observed.

#### 3.1.6. SIRT1 and Acetylated H1

SIRT1 directly interacts with the linker histone protein H1 [17], which binds to linker DNA to result in chromatin compaction [37]. H1 involvement in chromatin structure is also associated with cellular aging [38]. SIRT1 has been demonstrated to catalyze the deacetylation of histone 1 at lysine 26 (H1K26Ac) [17]. The authors speculate that this may further promote chromatin compaction. A separate study found that the ubiquitylation of H1 at lysine 64 may counteract this deacetylase activity, acting as a molecular switch [39]. SIRT1 was also observed to have deacetylase activity toward acetylated lysine 64 itself, as well as acetylated lysine 17 [40]. Deacetylation of both residues were observed to result in chromatin compaction. Table 1 is the summary of SIRT1 regulates the histone proteins described above, the other published data were summarized as Supplementary Table S1.

**Table 1:** SIRT1 regulates histone proteins.

Protein	Function	Activation of SIRT1	Inhibition of SIRT1	Key References
H3K9	Regulates gene silencing and heterochromatin formation.	Transcriptional repression and chromatin condensation.	Transcriptional activation and chromatin relaxation.	[19,20,21,22,23,24,25]
H3K14	Involved in transcriptional activation.	Gene silencing and chromatin compaction.	Promotes transcriptional activation and open chromatin.	[20,26,27]
H3K56	Important for DNA repair and chromatin remodeling.	Promotes chromatin stability and DNA repair mechanisms.	Loss of DNA repair function and chromatin instability.	[28,29,30,31]
H4K16	Critical for chromatin structure and gene regulation.	Chromatin condensation, gene silencing, and repression of transcription.	Chromatin relaxation and potential transcriptional activation.	[13,14,15,16,17,18]
H2AX	Involved in DNA damage response and repair.	Promote chromatin stability and facilitate DNA repair.	Impair DNA damage repair and might lead to genomic instability.	[32,33,34,35,36]
H1	Compacts chromatin and represses gene transcription	Promotes chromatin compaction and transcriptional silencing.	Gene activation, genomic instability.	[17,38,39,40]

### 3.2. SIRT1 Regulates Non-Histone Proteins

SIRT1 (Sirtuin 1) plays a critical role in regulating numerous cellular processes, including metabolism, stress response, aging, and gene expression. While SIRT1 is primarily known for deacetylating histones, it also regulates various non-histone proteins, influencing a broad range of cellular functions such as transcription, cell cycle control, DNA repair, and apoptosis.

Key non-histone proteins regulated by SIRT1 include those described below.

#### 3.2.1. SIRT1 and p53

p53 is a key tumor suppressor that promotes apoptosis, cell cycle arrest, and DNA repair in response to cellular stress or DNA damage. SIRT1, a NAD^+^-dependent deacetylase, regulates p53 by removing its acetyl groups, which weakens p53’s tumor-suppressive activity and reduces the transcription of pro-apoptotic genes. This deacetylation allows cancer cells to evade apoptosis and enhances their survival. The inhibition of p53 by SIRT1 is especially important in cancers with already compromised p53 pathways [41,42].

In breast cancer cells, for instance, SIRT1 deacetylates p53 at lysine 382, impairing the activation of downstream targets such as Bax and Puma, thereby facilitating stress tolerance and tumor progression [43]. In ovarian cancer, SIRT1 similarly deacetylates p53, reducing its activity in response to DNA damage. This results in the downregulation of p53 target genes, such as p21 (cell cycle arrest) and Bax (resulting in apoptosis), allowing the cancer cells to continue dividing despite DNA damage [44]. In colon cancer, SIRT1 deacetylates p53, impairing its ability to activate apoptotic pathways and allowing cancer cells to evade p53-dependent cell death [45]. In acute myeloid leukemia (AML), SIRT1 is upregulated and interacts with p53 to inhibit its function by deacetylating multiple lysine residues, thereby preventing apoptosis in response to DNA damage or oxidative stress [46]. Similarly, in non-small cell lung cancer (NSCLC), SIRT1 deacetylates p53 at lysine 382, diminishing its tumor-suppressive activity and enabling tumor cells to survive and proliferate despite genomic instability [47].

Although the underlying mechanism, SIRT1-mediated deacetylation of p53, is consistent, its impact differs among cancer types. In breast and ovarian cancers, SIRT1 often correlates with aggressive behavior and therapy resistance, while in colon cancer, its role appears more context-dependent, sometimes contributing to genomic stability under specific conditions. These variations highlight that the consequences of SIRT1-p53 interaction are influenced by tissue type, tumor stage, and cellular context.

In summary, in various cancers, SIRT1 deacetylates p53, suppressing its tumor-suppressive activity. By reducing p53’s ability to induce apoptosis, arrest the cell cycle, and regulate DNA repair, SIRT1 contributes to cancer cell survival, proliferation, and resistance to therapy. This mechanism makes SIRT1 a promising target for cancer therapies aimed at reactivating p53’s tumor-suppressive functions.

#### 3.2.2. SIRT1 and FOXO

The FOXO (Forkhead box O) transcription factors—mainly FOXO1, FOXO3a, FOXO4, and FOXO6—are key tumor suppressors in many cancers. They regulate genes involved in cell cycle arrest, apoptosis, DNA repair, metabolism, oxidative stress resistance, and immune modulation. In cancer, FOXO proteins often become inactivated through post-translational modifications (e.g., phosphorylation, acetylation), cytoplasmic sequestration, or degradation, enabling unchecked tumor growth [48].

SIRT1 regulation of FOXO transcription factors plays a complex and context-dependent role in breast and gynecological cancers (ovarian, endometrial, cervical). In these malignancies, the SIRT1–FOXO axis influences tumor cell survival, proliferation, apoptosis, and chemoresistance, depending on the balance between tumor-suppressive and pro-survival signaling [49]. Updated studies demonstrated that SIRT1 deacetylated FOXO proteins, acting as a tumor activator. For instance, SIRT1 was observed to deacetylate FOXO3a to alter its activity to favor cell survival under chemotherapy, resulting in chemoresistance and reduced apoptosis in breast cancer cells [50]. In certain gynecological cancers, SIRT1 deacetylated both FOXO1 and FOXO3a, reducing their pro-apoptotic and anti-proliferative effects and increasing proliferation and survival [51].

#### 3.2.3. SIRT1 and NF-κB

NF-κB (Nuclear Factor kappa-light-chain-enhancer of activated B cells) is a family of transcription factors that play critical roles in various cellular processes. It acts as a master regulator of immune response, inflammation, cell survival, and more. SIRT1 directly deacetylates the p65/RelA subunit of NF-κB at lysine 310, reducing its transcriptional activity [52]. Furthermore, small-molecule SIRT1 activators enhance p65 deacetylation, reducing TNFα-driven NF-κB activity and inflammatory cytokine release [53]. By deacetylating p65, SIRT1 represses the transcription of NF-κB target genes such as IL-6, TNF-α, and COX-2. Through this suppression, SIRT1 contributes to anti-inflammatory effects and may suppress tumorigenesis in certain contexts [54]. In aging and metabolic stress, reduced SIRT1 activity leads to increased NF-κB signaling and inflammation [52]. However, in some conditions, high SIRT1 boosts survival of metastatic breast cancer cells by suppressing NF-κB activity and oxidative stress [55].

#### 3.2.4. SIRT1 as an Activator of Non-Histone Proteins

Non-histone proteins are not always suppressed after deacetylation by SIRT1. For instance, when the transcription factor PGC-1α, which serves as a master regulator of mitochondrial proliferation and metabolism, is deacetylated, its transcriptional coactivation potential is enhanced, leading to increased binding to its target transcription factors, including NRF1, NRF2, PPARγ, and ERRα [56,57]. Additionally, deacetylation improves the stability of PGC-1α, preventing its ubiquitination and subsequent degradation [58]. Another example is the FOXO family mentioned above, including FOXO1, FOXO3a and FOXO4 proteins. Here, the deacetylation by SIRT1 enhances their DNA-binding, nuclear localization, and transcriptional activity, thereby increasing the expression of genes for antioxidant defense (e.g., MnSOD), cell cycle arrest, autophagy, and stress resistance [59]. Furthermore, when SIRT1 deacetylates the Ku70 DNA repair protein, the DNA-end binding and repair capacity of Ku70 is enhanced, improving the repair of double-strand breaks (e.g., after radiation). This also facilitates the suppression of apoptosis by sequestering the pro-apoptotic Bax protein. Therefore, by enhancing the function of Ku70, SIRT1 promotes DNA repair after radiation and reduces cell death [60].

Table 2 summarizes the described non-histone proteins deacetylated by SIRT1 and their activities if SIRT1 is activated or suppressed, and Supplementary Table S2 demonstrates the wide range of non-histone deacetylation targets, which are involved in regulating various cellular processes, including tumor suppression, metabolism, stress response, DNA repair, and circadian rhythm. SIRT1 plays a central role in these processes, either activating or suppressing the proteins’ functions depending on the cellular context and environmental cues.

### 3.3. SIRT1 Deacetylase Activity Is Modulated by Certain Conditions

The deacetylation activity of SIRT1 is complex and can be impaired under certain conditions.

NAD+ Depletion: SIRT1 requires NAD^+^ as a coenzyme. Conditions like oxidative or genotoxic stress activate PARP1, consuming NAD^+^ and thereby reducing SIRT1 activity [61]. Other cellular states that cause reduced NAD^+^ levels, such as aging, metabolic diseases, or stress, can similarly impair its deacetylase function [62].

Post-Translational Modifications of SIRT1: In some conditions, SIRT1 can be degraded. Oxidative stress, carbonylation, S-nitrosylation, S-glutathionylation from cigarette smoke, or reactive oxygen species (ROS) can inactivate SIRT1 and promote its degradation. Additionally, phosphorylation has been shown to reduce the stability of SIRT1 and impair its deacetylase function [63].

Altered Protein Interactions: In some instances, the binding of cofactors or other partners to SIRT1 may prevent it from interacting with its target proteins, thereby inhibiting its activity [64].

Cellular Context: Changes in cellular pathways (such as insulin resistance, inflammation, or mutations associated with cancer) may alter how SIRT1 interacts with its targets or prevent its deacetylase activity [65].

## 4. SIRT1 in Breast Cancer

Breast cancer is the most common cancer in women worldwide. In 2020, breast cancer surpassed lung cancer as the most frequently diagnosed cancer globally [66]. The classifications of breast cancer are commonly used as Receptor Status-Based by Immunohistochemistry (IHC), that is Hormone receptor-positive (ER^+^ and/or PR^+^), HER2-positive and Triple-negative (ER^−^, PR^−^, HER2^−^), as well as Molecular Subtype Classification (Luminal A/B, HER2-enriched and Basal-like) [67]. In breast cancer, SIRT1 can act both as a promoter and a depressor of cancer progression, depending on the context of its expression and the cellular environment [68,69,70]. Its role is complex because SIRT1 is involved in various cellular processes like cell survival, DNA repair [71], apoptosis, and metabolism. Its impact on breast cancer depends on how it interacts with key signaling pathways, tumor suppressors, and oncogenes [72].

The role of SIRT1 varies across different breast cancer subtypes:

### 4.1. SIRT1 in Hormone Receptor-Positive (HR^+^) Breast Cancer

In HR^+^ breast cancers, SIRT1 is often overexpressed and plays a pivotal role in tumor progression [73]. SIRT1 physically interacts with ERα, enhancing its transcriptional activity [74,75]. This interaction promotes the expression of antioxidant and pro-survival genes, such as catalase and glutathione peroxidase, while inhibiting tumor suppressor genes like cyclin G2 and p53. Consequently, SIRT1 activation supports estrogen-induced breast cancer growth and survival [73]. Notably, SIRT1 is overexpressed in ER^+^ breast cancer cases and regulates TGF-β, suggesting a role in tumor progression [75,76]. 

Given its role in promoting tumor survival and progression, SIRT1 is being explored as a potential therapeutic target [74]. For example, tamoxifen resistance is a significant challenge in ER^+^ breast cancer treatment. Studies have shown that SIRT1 is upregulated in tamoxifen-resistant breast cancer cells. This upregulation is associated with poor prognosis in tamoxifen-treated patients. Furthermore, the interaction between SIRT1 and Src kinase has been implicated in promoting tamoxifen resistance, highlighting SIRT1 as a potential therapeutic target [77,78].

Furthermore, obesity is a known risk factor for HR^+^ breast cancer. Prostaglandin E_2_ (PGE_2_), elevated in obesity, down-regulates SIRT1, leading to increased aromatase expression and estrogen production in adipose tissue. This demonstrates the complex role of SIRT1 in modulating link between obesity and breast cancer through hormone regulation [79]. Inhibition of SIRT1 has been shown to reduce estrogen-induced cell growth and tumor development, indicating that combining SIRT1 inhibitors with antiestrogen therapies could offer more effective treatment strategies for HR^+^ breast cancer [73].

### 4.2. SIRT1 in HER2-Positive (HER2^+^) Breast Cancer

HER2^+^ breast cancers are characterized by the overexpression of the HER2 receptor and are often more aggressive. SIRT1 is significantly overexpressed in HER2-enriched breast cancer subtypes compared to normal tissues. This overexpression is associated with increased tumor aggressiveness, suggesting that SIRT1 may contribute to the malignant phenotype in HER2^+^ tumors [73]. Moreover, high nuclear SIRT1 expression has been observed to be associated with reduced disease-specific survival, indicating a possible oncogenic role [80]. In HER2^+^ breast cancer cells, SIRT1 expression is induced by 17-β-estradiol through the G-protein-coupled estrogen receptor (GPER). This induction activates the EGFR/ERK/c-fos/AP-1 signaling pathway, promoting cell survival and resistance to DNA damage. Silencing GPER or SIRT1, or introducing the SIRT1 inhibitor Sirtinol, abolishes these effects, suggesting the importance of the SIRT1-GPER axis in HER2^+^ breast cancer progression [81].

However, a study analyzing 427 invasive ductal carcinoma cases found that higher SIRT1 expression in HER2^+^ tumors correlated with a lower risk of axillary lymph node metastasis (LNM), suggesting a potential tumor-suppressive role in this specific subtype [80]. These findings suggest that SIRT1’s role in HER2^+^ breast cancer may be context-dependent, potentially influenced by factors such as subcellular localization and interaction with other molecular pathways [82].

SIRT1 has been implicated in modulating the sensitivity of HER2^+^ breast cancer cells to targeted therapies. One study described how neratinib, a pan-HER tyrosine kinase inhibitor, induced senescence in HER2^+^ breast cancer cells (AU565 cell line) by increasing mitochondrial reactive oxygen species (ROS) and DNA damage. This process involved the downregulation of SIRT1, leading to the activation of p53 and p21 pathways [83]. In HER2^+^ breast cancer cells, SIRT1, along with SIRT6, has been shown to modulate the acetylation status of the transcription factor FOXO3, thereby influencing sensitivity to lapatinib, another HER2-targeted therapy. Inhibition of SIRT1/6 increased FOXO3 acetylation, enhancing its transcriptional activity and potentially sensitizing cells to lapatinib [84]. Overexpression of SIRT1 was found to counteract neratinib-induced senescence, suggesting that SIRT1 may confer resistance to neratinib by inhibiting senescence pathways. Quantitative analyses have shown that SIRT1 mRNA and protein levels are significantly higher in luminal A, luminal B (HER2−), and luminal B (HER2+) subtypes compared to matched normal tissues. This overexpression correlates with increased tumor aggressiveness, suggesting that SIRT1 may serve as a prognostic biomarker in these subtypes [77].

In summary, SIRT1 plays a complex role in HER2^+^ breast cancer, with evidence pointing to both tumor-suppressive and oncogenic functions depending on the context [85,86]. While its involvement in modulating response to HER2-targeted therapies like neratinib and lapatinib suggests its potential as a therapeutic target [84], further research is needed to fully elucidate SIRT1’s functions and to develop strategies for its modulation in HER2^+^ breast cancer treatment. Table 3 summarizes the role of SIRT1 in HR^+^ and HER2^+^ breast cancer. These findings demonstrate the complex and context-dependent role of SIRT1 in breast cancer. While it often promotes tumor progression in HR^+^ and HER2^+^ subtypes, its dual role under certain circumstances suggests that therapeutic strategies targeting SIRT1 should be carefully tailored to the specific molecular characteristics of the tumor (Table 3).

### 4.3. SIRT1 in Triple-Negative Breast Cancer (TNBC)

Triple-negative breast cancer (TNBC) is a subtype characterized by the absence of estrogen, progesterone, and HER2 receptors. Recent studies have explored SIRT1’s involvement in TNBC progression, metastasis, and therapeutic response. Like its activity in HER2^+^ breast cancer, SIRT1 exhibits complex and context-dependent roles in TNBC. While some studies suggest that SIRT1 functions as an oncogene, promoting tumor progression and metastasis [87], others indicate that its activation may suppress tumor cell proliferation through mechanisms involving mutant p53. Table 4 summarizes this dual role of SIRT1 in TNBC.

#### 4.3.1. The Oncogenic Role of SIRT1 in TNBC

Several studies have identified SIRT1 as a potential oncogene in TNBC.

SIRT1 expression and prognosis: In a study comparing TNBC to HR^+^ breast cancer subtypes, SIRT1 expression was significantly lower in TNBC. However, high SIRT1 expression in TNBC was associated with poor prognosis, suggesting a complex role in TNBC progression [88,89]. A clinical study conducted on 344 TNBC patients showed that SIRT1 overexpression correlates with tumor invasion, lymph node metastasis, and shorter disease-free survival. Functional experiments using siRNA also confirmed that silencing SIRT1 reduces invasiveness in TNBC cells [90].

Epithelial–Mesenchymal Transition (EMT) and Metastasis: SIRT1 has been implicated in promoting EMT in TNBC, a key process in cancer metastasis. SIRT1 induces EMT by modulating related pathways, contributing to increased tumor invasiveness. It can deacetylate and stabilize EMT-related transcription factors, thereby enhancing cell migration and invasion in TNBC via NF-κB signal pathways that involve stemness genes [91,92]. This high SIRT1 expression correlates with poor disease-free survival in TNBC patients [92]. Elevated SIRT1 levels are also associated with higher rates of lymph node metastasis, suggesting its role as a prognostic marker [90,93].

Cancer Stem Cell Properties: Inhibition of SIRT1 has been shown to reduce cancer stem cell populations and block EMT in breast cancer cells, indicating that SIRT1 contributes to the maintenance of stem-like properties and metastatic potential in TNBC [94].

#### 4.3.2. Tumor-Suppressive Role of SIRT1 in TNBC

In contrast, other research suggests that SIRT1 activation may suppress tumor progression in TNBC in certain contexts. One study found that low SIRT1 expression in TNBC was associated with worse overall survival, suggesting that SIRT1 may have a protective role in this subtype [88]. A separate study demonstrated that the SIRT1 activator YK-3-237 induced deacetylation of mutant p53 in TNBC cells, leading to decreased mutant p53 levels and upregulation of pro-apoptotic genes such as PUMA and NOXA. This resulted in reduced cell proliferation and induced cell cycle arrest at the G2/M phase [95]. Another study observed that SIRT1 expression is reduced in TNBC, and its loss alters the secretome of breast cancer cells, potentially influencing tumor behavior [96]. Jiang Y et al. [97] demonstrated that increasing NAD^+^ levels activate SIRT1, leading to the inhibition of EMT and metastasis in TNBC models. This effect is mediated through the deacetylation and inactivation of p66Shc, highlighting a potential therapeutic avenue [97] (Table 4).

**Table 4:** SIRT1’s Role in TNBC.

Study	Role of SIRT1	Mechanism	Outcome
Yi et al. (2013) [98]	Tumor-suppressive	Deacetylation of mutant p53	Reduced cell proliferation, G2/M arrest
Wang et al. (2022) [99]	Oncogenic	EMT promotion	Enhanced migration, poor prognosis
Zhang et al. (2015) [100]	Oncogenic	Cancer stem cell maintenance	Increased invasion, metastasis
Urra et al. (2018) [101]	Tumor-suppressive	AMPK activation	Reduced migration, increased survival

Therapeutic Implications

Given the dual roles of SIRT1 in TNBC, therapeutic strategies targeting SIRT1 must be approached with caution.

SIRT1 Inhibitors: Compounds like cambinol have shown efficacy in reducing cancer stem cell populations and blocking EMT in breast cancer cells. These inhibitors could potentially decrease tumor invasion and metastasis in TNBC [100,102]. Selisistat (EX-527), a selective SIRT1 inhibitor, enhances the efficacy of paclitaxel in TNBC models, suggesting a synergistic effect when combined with chemotherapy [103].

SIRT1 Activators: Agents such as YK-3-237 may offer therapeutic benefits by promoting the deacetylation of mutant p53, leading to tumor suppression in TNBC [98].

Natural Compounds: Compounds such as Cyanidin-3-glucoside (C3G), or montelukast, induce mesenchymal-to-epithelial transition (MET) in TNBC cells by upregulating SIRT1 expression, thereby inhibiting cell migration and invasion [91,104].

The contrasting roles of SIRT1 in TNBC highlight the necessity for subtype-specific therapeutic approaches. Further research is essential to elucidate the precise mechanisms by which SIRT1 influences TNBC progression and to develop targeted therapies that can modulate its activity effectively. SIRT1 plays a complex role in TNBC, acting as both a promoter and suppressor of tumor progression depending on the context. Its modulation affects key processes such as EMT, metastasis, and response to therapy. Targeting SIRT1, either through inhibition or activation, holds promise for developing novel therapeutic strategies against TNBC.

## 5. Sirt1 in Gynecologic Malignancies

As in breast cancer, in the context of gynecologic malignancies (such as ovarian cancer, endometrial cancer and cervical cancer), SIRT1 has been implicated in both tumor suppression and tumor progression, depending on the cellular context. Below, we outline how SIRT1 functions in gynecologic cancers, including its potential role in cancer development, progression, and therapy.

### 5.1. SIRT1 in Endometrial Cancer

Endometrial cancer (EC), a malignancy of the uterine lining, is one of the most common gynecological cancers. SIRT1’s role in endometrial cancer can be dualistic, having both tumor-promoting and tumor-suppressive functions. EC is classified into two main types based on histological characteristics: Type I and Type II [105]. These types differ in their molecular features, prognosis, and the involvement of various proteins like SIRT1 (Table 5).

#### 5.1.1. SIRT1 in Type I EC

Type I endometrial cancer (EC) is often associated with estrogen exposure and typically shows endometrioid histology. These tumors commonly carry PTEN mutations and alterations in the PI3K/AKT pathway [106,107]. Type I EC generally has a good prognosis, especially when detected early, with estrogen playing a key role in tumor initiation and progression [108]. SIRT1 contributes to hormone-driven tumor growth by enhancing ERα-mediated transcription [109]. It also promotes tumor progression by deacetylating and inactivating FOXO1, which reduces FOXO1’s tumor-suppressive activity in regulating the cell cycle, apoptosis, and stress response [110,111]. As mentioned above, p53, a crucial tumor suppressor, is often found to be deacetylated and inactivated by SIRT1 in several cancers, preventing p53’s activation of pro-apoptotic genes. This has also been observed in Type I EC, which may allow EC cells to evade apoptosis, contributing to tumor progression. The deacetylation of p53 by SIRT1 also inhibits p53-mediated cell cycle arrest, further promoting tumor cell proliferation [112,113]. SIRT1 plays a role in chemoresistance, which presents a major obstacle in endometrial cancer treatment, particularly with drugs like cisplatin. The ability of SIRT1 to deacetylate key proteins such as p53 and NF-κB can influence the resistance of endometrial cancer cells to chemotherapy-induced apoptosis. For instance, studies have shown that when SIRT1 deacetylates p53, it reduces its pro-apoptotic function and promotes chemoresistance to cisplatin in endometrial cancer cells [114]. 

In addition to its oncogenic activities, SIRT1 also has the potential to act as a tumor suppressor in specific contexts, particularly in regulating DNA repair and maintaining genomic stability. In endometrial cancer cells, SIRT1 activation can facilitate DNA damage repair by interacting with Ku70, a protein involved in the DNA repair machinery. Deacetylating Ku70 promotes DNA double-strand break repair and contributes to genomic stability. Therefore, SIRT1 can suppress genomic instability, a hallmark of cancer, and potentially prevent the development of endometrial cancer [60]. One study described that SIRT1 was significantly overexpressed in type I EC compared to normal endometrial tissue, and this overexpression correlates with improved progression-free survival, suggesting a potential tumor-suppressive role in this subtype [108]. Moreover, SIRT1 expression positively correlates with β-Catenin and ARID1A, both known tumor suppressors, which may contribute to the favorable prognosis observed in patients with higher SIRT1 levels [108].

#### 5.1.2. SIRT1 in Type II EC

In contrast, Type II EC is associated with serous or clear cell histology and non-estrogen-driven pathways. These tumors are typically characterized by mutations in p53 and alterations in pathways like PI3K/AKT/mTOR [115], as well as DNA repair mechanisms [107,116]. Type II EC generally has a poorer prognosis, with a higher likelihood of aggressive spread and a limited response to treatment, as non-hormonal carcinogenesis mechanisms play a more prominent role in this cancer type. In Type II EC, SIRT1 deacetylates both wild-type and mutant p53, but the consequences differ. Type II endometrial cancers, especially uterine serous carcinoma, frequently harbor missense mutations in TP53 [117]. These mutations not only disable p53’s tumor-suppressive function but also give it new abilities that drive tumor growth. Mutant p53 can increase cell proliferation, block apoptosis, and promote invasion and metastasis by altering gene expression [114]. In this setting, SIRT1 still deacetylates p53, but instead of suppressing it, SIRT1 stabilizes mutant p53, preventing its degradation (e.g., by MDM2) and strengthening its interactions with oncogenic partners such as NF-κB, HIF-1α, and c-Myc [118]. This stabilization amplifies the oncogenic potential of mutant p53, leading to enhanced chemoresistance (e.g., to paclitaxel and cisplatin), sustained DNA damage repair capacity, suppression of mitotic checkpoints, and promotion of tumor cell survival under genotoxic stress. Therefore, in type II EC with mutant p53, SIRT1 functions as a tumor promoter not by suppressing tumor suppression, but by amplifying oncogenic pathways driven by mutant p53 [118]. Mutant p53 exists in any type of cancer, most of them are somatic, and Olivier M [119] et al. discusses how TP53 mutations contribute to cancer by disrupting p53’s tumor-suppressing functions. It highlights their origins, impact on tumor behavior, and potential use in guiding diagnosis, prognosis, and treatment. In conclusion, the interaction between SIRT1 and p53 is a prime example of how molecular context dictates oncogenic behavior. In wild-type p53 tumors, SIRT1 inhibits tumor suppression by deacetylating and silencing p53. In contrast, in mutant p53-driven type II endometrial cancers, SIRT1 stabilizes and enhances the oncogenic functions of mutant p53, promoting tumor survival and chemoresistance.

The overexpression of SIRT1 enhanced resistance to cisplatin and paclitaxel in vitro and in vivo [114]. This is mediated through: support of DNA repair pathways, suppression of apoptotic signaling, and promotion of metabolic and survival adaptations [109].

SIRT1 was able to modulate cell cycles and DNA damage response. It deacetylated FOXO transcription factors, promoting survival under oxidative and genotoxic stress [49]; it also deacetylates Rb, E2F1, and other cell cycle regulators, promoting progression through S phase [120]. These activities enhance tolerance to DNA damage caused by chemotherapy and support the proliferation of genetically unstable cells [121].

On the other hand, in Type II EC, particularly clear-cell carcinoma, SIRT1 expression is generally lower than in type I EC. However, when expressed, it still correlates with better progression-free survival, indicating a nuanced role that may depend on specific tumor characteristics. At this point, SIRT1 exhibited tumor suppressor property; due to the limitation of study cases, the mechanisms need to be further clarified [110] (Table 5).

#### 5.1.3. Differences in SIRT1 Regulation Between Type I and Type II Endometrial Cancer

While SIRT1 acts as a key player in both Type I and Type II EC, its regulation and functional effects differ between the two subtypes, influencing tumorigenesis and cancer progression. Below we describe the key differences in SIRT1 regulation between Type I and Type II Ecs (summarized in Table 6).

Estrogen regulation and hormonal influence: Type I EC is often estrogen-driven, and SIRT1 regulation is heavily influenced by estrogen signaling. Estrogen activates estrogen receptors (ER), which can modulate the expression of SIRT1. Elevated levels of estrogen in Type I EC stimulate SIRT1 expression, promoting cell proliferation, survival, and metabolic regulation. This hormonal influence makes SIRT1’s role more prominent in the initiation and progression of Type I EC [122]. In contrast, Type II EC is estrogen-independent, with non-hormonal carcinogenesis mechanisms playing a more significant role. In this subtype, SIRT1 is not directly regulated by estrogen. Instead, its regulation is influenced by other factors such as DNA damage response pathways and cellular stress. Since Type II EC often lacks estrogen receptor expression, SIRT1 functions independently of estrogen signaling and contributes to tumor progression through alternative mechanisms, such as DNA repair and chemoresistance [108,114].

Role in DNA repair and chemoresistance: In Type I EC, SIRT1 helps maintain genomic stability by facilitating DNA repair processes, such as the repair of damage induced by estrogen-induced DNA addictions [123]. This repair activity can enhance the survival of cancer cells, contributing to chemoresistance in later stages of tumor progression. However, in early tumorigenesis, SIRT1’s regulation of tumor suppressors, such as p53, may help prevent uncontrolled cell growth, making it a dual regulator of both tumor suppression and promotion [114]. Meanwhile, in Type II EC, SIRT1 plays a more prominent role in DNA damage repair due to the aggressive and treatment-resistant nature of these tumors. Cisplatin and other chemotherapy agents often induce DNA damage in Type II EC cells, and SIRT1 aids in the repair of this damage, enhancing the cells’ resistance to chemotherapy [123]. p53 mutations in Type II tumors lead to the loss of a key tumor suppressor, and SIRT1 exacerbates this issue by deacetylating and inactivating p53, allowing tumor cells to escape apoptosis. In this context, SIRT1 is critical for chemoresistance and tumor progression [124].

Tumor suppressor and oncogenic pathways: In Type I EC, SIRT1 can act as both a tumor suppressor and an oncogene. In the early stages of tumorigenesis, SIRT1 may help activate tumor suppressors like p53, supporting tumor suppression and the prevention of excessive cell growth [123]. However, in more established tumors, SIRT1 has the ability to inactivate p53 and activate other oncogenic pathways like PI3K/AKT that contribute to tumor progression and chemoresistance [125]. In Type II EC, SIRT1’s activity is more aligned with promoting oncogenic pathways. SIRT1’s inhibition of p53 function exacerbates the tumorigenic effects of p53 mutations, allowing for unchecked cell survival and proliferation.

EMT and invasion: SIRT1 plays a role in promoting epithelial–mesenchymal transition (EMT) in both Type I and Type II endometrial cancers, though its impact is considerably more pronounced in Type II disease. In Type I EC, SIRT1 may contribute to enhanced cell migration and invasion by deacetylating EMT-related transcription factors such as Snail and Slug. However, this mechanism appears to be less central to tumor progression, reflecting the generally less aggressive nature of Type I tumors [125].

In contrast, EMT is a hallmark of Type II EC, and SIRT1 functions primarily as an oncogenic driver in this context. By deacetylating transcription factors including Snail and Slug, SIRT1 promotes the transition from an epithelial to a mesenchymal phenotype, thereby facilitating tumor cell invasion and metastasis [122]. This EMT-driven invasiveness contributes to the high metastatic potential and poor prognosis associated with Type II EC.

Mitochondrial function and autophagy: SIRT1’s regulation of mitochondrial function in Type I EC promotes cell survival under metabolic stress. By modulating mitochondrial dynamics and autophagy, SIRT1 helps cancer cells maintain energy homeostasis, facilitating their growth and survival. This regulation may support the expansion of Type I EC cells, particularly in the early stages [123]. In Type II EC, SIRT1’s role in mitophagy is similarly crucial for maintaining mitochondrial integrity, especially under stress conditions. By clearing dysfunctional mitochondria, SIRT1 supports cancer cell survival in the face of oxidative stress and metabolic challenges. This helps to sustain the aggressive nature of Type II EC, promoting tumor growth even in unfavorable environments [110].

In summary, the regulation of SIRT1 differs significantly between Type I and Type II ECs. In Type I EC, SIRT1 is influenced by estrogen signaling and plays a dual role in regulating tumor suppression and progression. It supports early tumorigenesis through the activation of tumor suppressors like p53, but its function shifts to promoting tumor progression and chemoresistance in later stages. In contrast, Type II EC is estrogen-independent, and SIRT1 is primarily regulated by DNA damage and stress responses. It plays a more oncogenic role in this subtype by promoting chemoresistance, EMT, and tumor invasion through the inactivation of p53 and regulation of key metastasis-related pathways.

### 5.2. SIRT1 in Ovarian Cancer

Histological classification of ovarian cancer by cell origin includes the following: epithelial ovarian cancer, which accounts for 85–90% of cases; germ cell tumors, which arise from reproductive cells; and sex cord-stromal tumors, which originate from the connective tissue and hormone-producing cells of the ovary [126,127]. As in the other cancers discussed thus far, SIRT1 plays a multifaceted role in tumor progression, metastasis, and drug resistance in ovarian cancer. The enzyme can either act as a tumor promoter or a tumor suppressor, depending on the cellular context and tumor microenvironment [128,129]. However, SIRT1 is frequently overexpressed in ovarian cancer tissues compared to normal ovarian epithelium. Elevated SIRT1 expression has been correlated with poor prognosis, aggressive tumor behavior, and resistance to chemotherapy. High SIRT1 levels are associated with reduced overall survival and increased tumor grade and stage [128,129].

#### 5.2.1. SIRT1 as a Tumor Promoter in Ovarian Cancer

In ovarian cancer, SIRT1’s deacetylation activity can promote cell survival, proliferation, and metastasis, which contributes to cancer progression [130]. It regulates stemness and tumor initiation and enhances the expression of stemness-related genes such as Nanog, SOX2, and OCT4, promoting cancer stem cell (CSC) phenotypes. This contributes to tumor initiation, metastasis, and therapeutic resistance [131,132]. SIRT1 also modulates oxidative stress responses by deacetylating FOXO3a, promoting survival under cytotoxic stress [133]. Furthermore, SIRT1 is involved in regulating chemoresistance, a major challenge in ovarian cancer treatment [134]. SIRT1 can enhance the survival of ovarian cancer cells by deacetylating and inactivating p53, a tumor suppressor that promotes apoptosis. This prevents p53-mediated apoptosis in response to chemotherapy, leading to drug resistance, particularly to cisplatin and paclitaxel [44,135,136]. Cisplatin resistance is one of the most common forms of chemoresistance in ovarian cancer, and studies have shown that SIRT1 overexpression in ovarian cancer cells enhances resistance to cisplatin treatment by reducing the activity of p53. Inhibition of SIRT1 sensitizes ovarian cancer cells to cisplatin and paclitaxel by reactivating apoptosis pathways [137,138]. Other pathways regulated by SIRT1 include the AMPK/SIRT1/PGC1α [139], AKT/FOXO3a [133], and PKM2/mTOR pathways [140]. Moreover, through deacetylation of p53 and Ku70, SIRT1 prevents apoptosis by downregulating pro-apoptotic genes and enhancing DNA repair mechanisms, thus supporting tumor cell survival [136,141].

#### 5.2.2. SIRT1 as a Tumor Suppressor in Ovarian Cancer

In some contexts, SIRT1 can also act as a tumor suppressor, particularly with regard to regulating apoptosis and DNA repair mechanisms. SIRT1 was observed to also deacetylate certain tumor activation factors such as Claudin 5, which resulted in suppressing ovarian cancer [142]. In certain settings, SIRT1 suppresses ovarian cancer depending on its subcellular localization and counters tumor growth [143,144]. However, these suppressive roles are less well-characterized in ovarian cancer compared to its oncogenic actions.

### 5.3. SIRT1 in Cervical Cancer

Cervical cancer remains one of the most common gynecological malignancies, with persistent infection by high-risk human papillomavirus (HPV), especially types 16 and 18, being the primary etiological factor.

In cervical cancer, SIRT1 plays a complex role that can either promote tumor progression or tumor suppression, depending on the cellular context. SIRT1 is significantly overexpressed in cervical cancer tissues compared to normal cervical epithelium. Elevated SIRT1 levels are associated with reduced overall survival and may serve as a prognostic biomarker [145,146]. Additionally, immunohistochemical analyses have shown increased nuclear and cytoplasmic SIRT1 expression in squamous intraepithelial lesions (SILs) and invasive squamous cell carcinomas (SCCs) [147]. Furthermore, the role of SIRT1 is particularly important in the context of HPV infection, with recent studies showing that SIRT1 interacts with HPV oncogenes and contributes to cervical tumorigenesis.

#### 5.3.1. SIRT1 and HPV Oncogenesis

HPV encodes two oncoproteins, E6 and E7, that interfere with tumor suppressors like p53 and Rb, driving the development of cervical cancer. SIRT1 has been shown to interact with E7 oncoprotein. This interaction leads to deacetylation and inactivation of p53, promoting cell survival and proliferation in HPV-positive cervical cancer cells. HPV E7 protein can also upregulate SIRT1 expression, which further enhances chemoresistance and cell proliferation. Furthermore, HPV E6 protein interacts with SIRT1 to further promote the degradation of p53. This allows cervical cancer cells to evade the tumor-suppressive effects of p53 and contributes to the survival and proliferation of cancer cells [148,149]. This suggests that SIRT1 plays a role in the transformation of cells infected by HPV, particularly by preventing apoptosis and promoting tumorigenesis.

Beyond E6 and E7, SIRT1 also promotes the fidelity of HPV16 E1-E2 DNA replication. The absence of SIRT1 leads to reduced recruitment of the DNA repair protein Werner helicase (WRN) to replicating viral DNA, compromising replication fidelity [150]. SIRT1 also interacts with the HPV16 E2 protein and TopBP1, forming a complex that regulates viral replication and the DNA damage response. This interaction facilitates the persistence of HPV infection, contributing to cervical carcinogenesis [148].

#### 5.3.2. SIRT1 Expression, Prognosis and Chemoresistance

High SIRT1 expression predicts a poor response to neoadjuvant chemotherapy (NAC) in locally advanced cervical cancer. Lastly, patients with lower SIRT1 levels exhibit better treatment outcomes, indicating its potential as a predictive biomarker [151,152]. SIRT1 expression increases progressively from normal cervical epithelium to low-grade and high-grade cervical intraepithelial neoplasia (CIN) and invasive squamous cell carcinoma (SCC), suggesting its involvement in cervical carcinogenesis [153,154]. SIRT1 contributes to chemoresistance in cervical cancer by modulating key signaling pathways such as β2-adrenergic receptor (β2-AR) signaling. Activation of β2-AR upregulates SIRT1 expression, which in turn deacetylates p53, reducing its tumor suppressor activity and leading to increased resistance to doxorubicin-induced apoptosis [155]. SIRT1 is also overexpressed in paclitaxel-resistant cervical cancer cells, and the knockdown of SIRT1 inhibits cell proliferation and promotes apoptosis, suggesting its role in mediating resistance [156].

#### 5.3.3. Immune Evasion and Inflammasome Suppression

In addition to its role in preventing apoptosis, recent literature suggests SIRT1 also plays a role in affecting pyroptosis. SIRT1 suppresses the expression of absent in melanoma 2 (AIM2), a component of the inflammasome complex, by destabilizing RELB mRNA. This repression prevents pyroptosis, allowing cervical cancer cells to evade immune-mediated cell death [146]. Research on the role of SIRT1 in modulating this form of programmed cell death is limited and may serve as a promising area for further investigation.

#### 5.3.4. SIRT1 and Tumor Suppression in Cervical Cancer

While SIRT1 is often considered to play a pro-tumorigenic role in cervical cancer, it can also function as a tumor suppressor under certain conditions. This duality in function may be dependent on cellular context and the balance of activating and inhibiting signals. For example, while the Fra-1 transcription factor tends to inhibit cervical cancer cell proliferation and promotes apoptosis at normal cellular concentrations, overexpression of Fra-1 leads to downregulation of SIRT1, suggesting that in this specific context, reduced SIRT1 levels contribute to tumor suppression [156].

In summary, SIRT1 plays an important role in the pathogenesis and progression of cervical cancer through its effects on cell proliferation, metastasis, chemoresistance, immune evasion, and viral replication. Its overexpression is associated with poor prognosis, and its inhibition presents a potential therapeutic strategy. Nevertheless, further research is warranted to fully elucidate SIRT1’s mechanisms and to develop targeted therapies for improved clinical outcomes in cervical cancer patients.

### 5.4. Therapeutic Potential of SIRT1 in Gynecologic Malignancies

SIRT1 has garnered significant attention in the context of gynecologic cancers—especially in ovarian, endometrial, and cervical cancers—due to its multifaceted roles in tumor progression, chemoresistance, and cellular metabolism. Therapeutic strategies targeting SIRT1 are under active investigation, with several preclinical studies highlighting its potential as a therapeutic target.

SIRT1 in Endometrial Cancer

SIRT1’s role in endometrial cancer (EC) appears to be context-dependent, with studies indicating both tumor-promoting and tumor-suppressing functions. Some studies have observed higher SIRT1 expression in EC tissues compared to normal endometrial tissues, suggesting a role in tumor progression [109,114]. SIRT1 has been implicated in the development of resistance to chemotherapy in EC. Inhibiting SIRT1 expression has been shown to enhance the sensitivity of EC cells to chemotherapeutic agents [111]. The long non-coding RNA FIRRE has been found to regulate SIRT1-mediated autophagy, affecting the radiation sensitivity of EC cells [151]. Targeting this pathway may offer new avenues for enhancing radiotherapy efficacy [152]. Given its dual role in endometrial cancer, SIRT1 is considered both a therapeutic target and a biomarker for predicting chemoresistance and prognosis. Targeting SIRT1 with inhibitors such as EX-527 or MHY2256 has shown promise in sensitizing endometrial cancer cells to chemotherapy and overcoming chemoresistance [114,153].

SIRT1 in Ovarian Cancer

In ovarian cancer, SIRT1 is often overexpressed, contributing to tumorigenesis, metastasis, and resistance to chemotherapy. Inhibition of SIRT1 has been shown to sensitize OC cells to cisplatin and paclitaxel by promoting apoptosis and reducing cell proliferation. Elevated SIRT1 levels have been associated with resistance to platinum-based chemotherapies. Research found that puerarin, a natural isoflavone, induces apoptosis in platinum-resistant epithelial ovarian cancer cells by targeting SIRT1. The study suggests that puerarin’s anticancer effects are mediated through the downregulation of SIRT1, leading to increased apoptosis in resistant cancer cells [134].

The novel SIRT1 inhibitor MHY2245 has demonstrated the ability to induce autophagy and inhibit energy metabolism in OC cells via the PKM2/mTOR pathway, leading to reduced tumor growth in preclinical models. MHY2245 was shown to inhibit SIRT1 activity, induce autophagy, and promote apoptosis in human ovarian cancer cells. The compound decreased SIRT1 expression and increased the expression of apoptotic factors such as caspase-3 and Bax, highlighting its potential as a therapeutic agent [153,157].

Puerarin, a natural isoflavone, has been reported to induce apoptosis in platinum-resistant OC cells by targeting SIRT1, suggesting potential for combination therapies [134]. Moreover, several Sirt1 inhibitors and compounds which suppress sirt1 expressions, included EX527, 15d-PGJ2, MHY2245 and Gerberoside were performed to treat ovarian cancer in vivo [154,157].

SIRT1 in Cervical Cancer

Research on SIRT1 in cervical cancer is less extensive, but emerging studies suggest its involvement in tumor progression and response to therapy [147,154]. Given its involvement in tumor progression, chemoresistance, and immune evasion, SIRT1 represents a promising therapeutic target. Inhibitors of SIRT1, such as EX527, or thiocyanates, have demonstrated efficacy in impairing cervical cancer cell growth and inducing cell death [147,158].

SIRT1 may contribute to cervical cancer development by modulating pathways related to cell proliferation and apoptosis [148,159]. While direct therapeutic interventions targeting SIRT1 in cervical cancer are still under investigation, its role in key cellular processes makes it a potential candidate for future therapies [160].

## 6. SIRT1 Inhibitors in Pre-Clinical Trials for Breast Cancer and Gynecological Malignances

SIRT1 plays a significant role in the progression and treatment resistance of various cancers, including breast and gynecological malignancies. Consequently, SIRT1 inhibitors have garnered attention as potential therapeutic agents. Below is an overview of notable SIRT1 inhibitors and their relevance to breast and gynecological cancers.

### 6.1. Selisistat (EX-527)

Selisistat is a potent and selective SIRT1 inhibitor. Initially developed for Huntington’s disease [161], Selisistat has been investigated for its potential in cancer therapy. Preclinical studies have demonstrated that Selisistat enhances the efficacy of paclitaxel in both luminal and triple-negative breast cancer models by sensitizing cancer cells to apoptosis [103]. 

### 6.2. MHY2245

MHY2245 is a synthetic inhibitor of SIRT1 that triggers both autophagy and apoptosis in cancer cells. In ovarian cancer cell lines, MHY2245 demonstrated strong cytotoxic effects, exceeding those of doxorubicin. It suppressed SIRT1 activity, caused cell cycle arrest at the G2/M phase, and induced apoptotic cell death. In vivo experiments showed that MHY2245 significantly inhibited tumor growth in xenograft models; specifically, tumor growth in nude mice bearing SKOV3-derived tumors was markedly reduced by MHY2245 administered intraperitoneally at doses of 0.5 and 1 mg/kg/day [153].

### 6.3. Combinol (SIRT1/2 Inhibitor)

Cambinol was first identified as a SIRT1/2 inhibitor (IC_50_ ≈ 50–60 μM) with anti-tumor activity in preclinical lymphoma and carcinoma models. It reduces tumor cell viability and induces apoptosis by increasing acetyl-p53 and FOXO3a levels [162]. At 50 μM in breast cancer MCF-7 and other cell lines, Cambinol decreases aromatase expression and inhibits proliferation. In breast cancer models, combining Cambinol with paclitaxel shows additive-to-synergistic antiproliferative effects [103]. A dose of 100 mg/kg was used in mice without apparent toxicity (no significant weight loss or increases in serum transaminases) in some studies [163]. 

### 6.4. Sirtuin 1 Inhibiting Thiocyanates (S1th)

S1th compounds represent a new class of isotype-specific SIRT1 inhibitors [158]. In cervical cancer cells, S1th compounds induced cell death, highlighting their potential as therapeutic agents in gynecological malignancies. Currently, S1th compounds are in preclinical in vitro stages; no in vivo or clinical trial data (animals or humans) are published for S1th. The IC_50_ values are in the micromolar range, which shows modest potency compared to some other inhibitors (e.g., EX-527), so further optimization is required for better potency, bioavailability, and pharmacokinetic properties.

Despite the mechanistic link, direct confirmation of SIRT1 activation in vivo remains limited, as most trials do not assess SIRT1 activity or expression as a biomarker. Future studies need to address this gap and evaluate whether NAD^+^ repletion leads to meaningful and sustained activation of SIRT1 in disease-relevant tissues.

## 7. From Preclinical to Clinical Trials

In the past five years, there have been no large, fully randomized oncology trials specifically testing selective SIRT1 inhibitors or activators in breast or gynecologic cancers. Human studies have primarily focused on indirect SIRT1 modulators-such as resveratrol and nicotinamide/NAD^+^ boosters-or have reported safety and pharmacological data for SIRT1 modulators investigated in non-oncology settings. However, several of these compounds have been used in clinical trials for the treatment of other diseases.

### 7.1. SRT2104 (SIRT1 Activator)

SRT2104 is a selective activator of SIRT1 that has been studied in humans for metabolic, inflammatory, and age-related conditions. Human trials (up to the year 2020) indicate that it is generally safe and well-tolerated, with modest metabolic benefits such as improvements in lipid profiles and mitochondrial function [164,165], though no consistent effects on glycemic control have been observed [166]. However, pharmacokinetic variability and inconsistent absorption remain challenges [164]. To date, there are no published clinical cancer trials involving SRT2104. While preclinical evidence suggests potential benefits in diseases where SIRT1 activation may be advantageous, its translation into oncology remains unproven [164,165,166,167,168].

### 7.2. EX-527 (Selisistat, SIRT1 Inhibitor)

Although EX-527 has been widely used as a research tool in cellular and animal studies, its clinical translation remains relatively limited. Key human trials and findings are summarized below. EX-527 has demonstrated good short-term safety and tolerability in both healthy volunteers and patients with Huntington’s disease (HD), with single doses up to 600 mg and daily doses of 300 mg for seven days being well tolerated and associated with minimal adverse effects [161]. Its pharmacokinetics are reasonably well characterized, showing predictable absorption, a modest half-life, and moderate inter-subject variability (~46% coefficient of variation) [169]. However, clinical trials to date have been short in duration (up to 14 days), exploratory in nature, conducted at oral doses ranging from 10 to 100 mg/day, and focused primarily on biomarker endpoints rather than therapeutic efficacy [169]. No significant changes were observed in biomarkers such as soluble huntingtin [161], and currently, there is no published clinical data on the use of EX-527 in cancer or other chronic disease settings. Long-term safety, dose optimization, and translational potential remain largely unexplored, and a withdrawn reproductive trial underscores the ongoing challenges in advancing its clinical application.

### 7.3. Resveratrol (SIRT1 Activator)

Resveratrol is a naturally occurring polyphenol found in red grapes, berries, and peanuts, best known for its antioxidant and anti-inflammatory properties [170]. By activating SIRT1, resveratrol may mimic some effects of caloric restriction, potentially promoting cellular longevity and mitochondrial health. Resveratrol has been studied in numerous human clinical trials and is generally considered safe and well tolerated, even at high doses up to 5 g/day. Most adverse effects are mild and gastrointestinal in nature. However, its clinical efficacy remains inconsistent across studies. In cardiovascular and metabolic settings, some trials report modest benefits in vascular function and insulin sensitivity, particularly in older or at-risk adults], while others show no significant changes in lipid profiles, glucose control, or weight [171]. In cancer prevention trials, resveratrol has been shown to reach target tissues such as the liver and may modulate signaling pathways like Wnt, but no definitive therapeutic effects have been demonstrated [172]. A major limitation is its poor oral bioavailability due to rapid metabolism, which hinders systemic exposure to the active compound [170]. Most trials to date have been short in duration, small, or focused on prevention rather than treatment, limiting the strength of clinical conclusions [171].

### 7.4. Nicotinamide Adenine Dinucleotide (NAD^+^)

Nicotinamide adenine dinucleotide (NAD^+^) is a vital coenzyme that serves as an essential substrate for sirtuins, particularly SIRT1. Its activity is tightly linked to intracellular NAD^+^ levels, meaning that NAD^+^ availability directly controls the enzymatic function of SIRT1 [1,2,3,4]. Boosting NAD^+^ through supplementation with precursors such as nicotinamide riboside (NR) or nicotinamide mononucleotide (NMN) is therefore hypothesized to enhance SIRT1 activity and its downstream effects. The highest well-tolerated dose of NR reported in a human trial is 3000 mg/day (administered as 1500 mg twice daily) over a period of four weeks, without serious adverse effects in the study population [173]. In clinical trials, oral supplementation with NR or NMN has been shown to be safe and well tolerated at doses of up to 1000 mg/day in Parkinson’s disease (PD) [174], consistently increasing blood NAD^+^ levels in humans. However, clinical benefits remain variable [173]. In insulin-resistant individuals and patients with type 2 diabetes, NR did not improve insulin sensitivity or glycemic control [175]. In contrast, older adults with peripheral artery disease (PAD) or mild muscle injury showed modest improvements in physical performance and muscle regeneration, which may be mediated in part by increased SIRT1 activity [176].

### 7.5. Panobinostat (LBH589)

Panobinostat is a pan-HDAC inhibitor with activity against multiple HDAC enzymes, including SIRT1 but not specifically targeting it. Panobinostat has been evaluated in clinical trials for various malignancies, including breast cancer. Preclinical studies have shown that Panobinostat can selectively target triple-negative breast cancer cells by inducing hyperacetylation and cell cycle arrest at the G2-M DNA damage checkpoint [177]. In human studies, 20 mg oral dosing leads to C_max_ ~21.6 ng/mL (~62 nM) approximately 1 h post-dose [178]. In multiple myeloma clinical PK/PD studies (B2207), the geometric mean C_max_ was ~8.1 ng/mL (~23 nM) when used with bortezomib/dexamethasone [179].

### 7.6. The Gap and Challenge from Preclinical to Clinical

Despite strong preclinical evidence supporting the role of SIRT1 in regulating metabolism, aging, inflammation, and neurodegeneration, the clinical translation of SIRT1-targeted therapies has faced significant challenges. A major issue is pharmacokinetic variability and low bioavailability; compounds such as SRT2104 and resveratrol exhibit suboptimal absorption, short half-lives, and high inter-individual variability, which limit consistent target engagement in humans [164,165,166,167,168,169,170,171,172]. Additionally, several early SIRT1 activators, including SRT1720 and resveratrol, were later found to activate SIRT1 only in artificial assay systems, raising concerns about off-target effects and uncertain mechanisms. Clinical trials have generally been short in duration (7–28 days) and focused on safety or biomarker endpoints rather than long-term disease modification, limiting their relevance for chronic conditions like type 2 diabetes or neurodegeneration. Moreover, target engagement is often not confirmed in vivo; for example, EX-527 showed no significant changes in soluble huntingtin or inflammatory markers in a Huntington’s disease trial despite achieving expected plasma concentrations [161].

Compounding these issues, the context-dependent biology of SIRT1—acting as a tumor suppressor in some settings and a promoter of tumor survival in others—adds complexity and risk, particularly in oncology [11]. These challenges underscore the need for more selective and bioavailable SIRT1 modulators, validated in vivo biomarkers, and well-designed, disease-specific trials to fully realize the therapeutic potential of SIRT1 modulation in human disease. While several SIRT1 inhibitors have shown promise in preclinical studies for breast and gynecological cancers, clinical trials specifically targeting these malignancies remain limited. Existing data highlights the potential of SIRT1 inhibitors as adjuncts to conventional therapies, particularly in overcoming chemoresistance. Further clinical investigations are warranted to validate their efficacy and safety in cancer patients.

## 8. Conclusions

SIRT1 (Sirtuin 1) is an NAD^+^-dependent deacetylase that regulates multiple non-histone proteins (e.g., p53, FOXO, NF-κB) and pathways involved in DNA repair, cell cycle, apoptosis, metabolism, and epigenetic remodeling. Its role in breast cancer and gynecologic malignancies is context-dependent, with it functioning either as a tumor promoter or suppressor depending on the cancer subtype, stage, and molecular environment. While SIRT1 may support DNA repair and tumor suppression in early stages, it more commonly promotes tumor progression, metastasis, and therapy resistance in advanced disease. Targeting SIRT1 offers a promising strategy to improve cancer treatment outcomes, particularly in chemoresistant subtypes. From a therapeutic perspective, it presents a novel approach to enhancing treatment responses, especially in cancers with poor prognosis or resistance to standard therapies. However, translating these findings from preclinical models to clinical settings remains challenging. Overcoming these hurdles will require the development of more selective and potent compounds, improved patient stratification using molecular markers, and well-designed, longer-term clinical studies to fully realize the potential of SIRT1-targeted therapies in breast and gynecologic cancers.

## Figures and Tables

**Table 2:** SIRT1 regulates non-histone proteins.

Protein	When Deacetylated	Effect	Activation/Suppression	Main Function	Key References
P53	Decreased apoptosis and Increased survival	Inhibited transcriptional activity	Suppression	Tumor suppressor	[41,42,43,44,45,46,47]
FOXO	Oxidative stress, nutrient deprivation	Enhanced transcriptional activity	Activation	Cellular response to oxidative stress, metabolism, and longevity	[48,49,50,51]
NF-κB	Inflammation, oxidative stress	Inhibited transcriptional activity	Suppression	Inflammation, immune response, cell survival	[52,53,54,55]
PGC-1α	Exercise, metabolic stress	Enhanced mitochondrial function	Activation	Mitochondrial biogenesis, oxidative metabolism	[56,57,58]
Ku70	DNA damage, genotoxic stress	Enhanced DNA repair function	Activation	DNA repair, genomic stability	[60]

**Table 3:** SIRT1’s Role in HR^+^ and HER2^+^ Breast Cancer.

Subtype	SIRT1 Expression	Role in Tumorigenesis	Therapeutic Implications
HR^+^ (ER^+^/PR^+^)	Overexpressed	Promotes tumor growth via ERα interaction	Targeting SIRT1 may enhance antiestrogen therapy efficacy
HER2^+^	Overexpressed	Supports survival via GPER-mediated signaling	SIRT1 inhibition could sensitize to DNA-damaging agents

**Table 5:** Overview of Endometrial Cancer Subtypes.

Feature	Type I EC	Type II EC
Hormone dependence	Estrogen-dependent	Estrogen-independent
Histology	Endometrioid	Serous, Clear cell
Genetic alterations	PTEN, PI3K/AKT, KRAS, ARID1A	TP53 mutation, chromosomal instability
Grade/Prognosis	Low-grade, better prognosis	High-grade, aggressive, poor prognosis

**Table 6:** SIRT1 Functional Differences in Type I vs. Type II EC.

Aspect	Type I EC	Type II EC
Role in Tumor Biology	Tumor promoter	Dual role: tumor promoter and DNA repair mediator
Hormone pathway interaction	Enhances ERα activity, promotes estrogen-mediated proliferation	Minimal role in hormone signaling
P53 interaction	Deacetylates and inactivates wild-type p53, reducing apoptosis	Deacetylates mutant p53, modulating DNA damage response
FOXO1 regulation	Inactivates FOXO1, reducing cell cycle arrest and apoptosis	Less studied, but likely similar suppression
DNA repair function	Modest enhancement of repair (via Ku70, PARP1)	Strong enhancement of HR/NHEJ, promotes genomic survival
Chemoresistance role	Promotes resistance to cisplatin, progestins by inhibiting apoptosis	Promotes resistance to paclitaxel/carboplatin by enhancing DNA repair
Stemness and EMT promotion	Increases expression of stemness/EMT markers via Wnt/Notch/β-catenin pathways	May support EMT and invasion, but driven more by genomic instability
Therapeutic relevance	Targeting SIRT1 can restore hormone/chemo sensitivity	SIRT1 inhibitors may sensitize to chemo, especially in p53-mutant tumors

## Data Availability

Data are contained within the article and Supplementary Materials.

## Supplementary Materials

The following supporting information can be downloaded at: https://www.mdpi.com/article/10.3390/biology14111510/s1. Table S1: SIRT1 regulates histone proteins; Table S2: SIRT1 regulates non-histone proteins [180,181,182,183,184,185,186,187,188,189,190,191].
