# Supplementary material for: The Roles of Sirt1 in Breast and Gynecologic Malignancies
Source: Biology (Basel). 2025 Oct 28;14(11):1510. doi: 10.3390/biology14111510 (PMC12650002; doi:10.3390/biology14111510)
Supplement: Supplementary file 1 [file biology-14-01510-s001.zip › biology-3909039-supplementary.pdf]

Table S1. SIRT1 regulates histone proteins

Protein	Function	Activation of SIRT1	Inhibition of SIRT1	Key references
**H3K27**	Linked to active transcription and enhancers.	Transcriptional repression and chromatin compaction.	Increased acetylation and activation of transcription.	[1-4]
**H3K18**	Associated with transcriptional activation.	Transcriptional repression.	Chromatin relaxation and gene activation.	[1-4]
**H4K20**	Associated with transcriptional regulation and DNA repair.	Transcriptional repression.	Promotes transcriptional activation.	[5,6]
**H2AK5**	Associated with transcriptional activation and chromatin remodeling.	Chromatin compaction and transcriptional repression.	Chromatin relaxation and potential transcriptional activation.	[7-11]
**H2AK9**	Linked to transcriptional regulation and chromatin structure.	Transcriptional repression and chromatin condensation.	May be associated with gene activation and chromatin relaxation.	[7-11]
**MacroH2A**	Function of Modification	Effect of SIRT1 Deacetylation	Effect of SIRT1 Inhibition	[12-15]
**Macro H2A1.1K152**	Involved in gene silencing, X-inactivation, and chromatin condensation.	Transcriptional repression and chromatin compaction, reinforcing gene silencing.	May promote chromatin relaxation and gene activation.	[12-15]
**H2BK5**	Associated with transcriptional activation and chromatin remodeling.	Chromatin compaction and transcriptional repression.	May result in gene activation and chromatin relaxation.	[12-15]
**H2BK12**	Linked to transcription regulation and chromatin structure.	Transcriptional repression and chromatin condensation.	Could correlate with increased transcription and chromatin de-condensation.	[12-15]

Table S2. SIRT1 regulates non-histone proteins

Protein	When deacetylated	Effect	Activation/ Suppression	Main function	Key references
**HIF-1α**	Hypoxia, low oxygen availability	Increased stability and transcription al activity	Activation	Cellular response to hypoxia, oxygen sensing	[16]
**LKB1**	Nutrient stress, metabolic cues	Enhanced AMPK activation, energy regulation	Activation	Energy homeostasis, AMPK signaling	[17]
**E2F1**	Cell cycle, DNA damage	Affects cell cycle progression, apoptosis	Activation	Cell cycle progression, apoptosis regulation	[18]
**STAT3**	Cytokine signaling, stress	Influences transcription al activity	Activation	Cytokine signaling, immune response, cancer	[19]
**IRS2**	Insulin signaling, glucose metabolism	Regulates insulin signaling and glucose metabolism	Activation	Insulin signaling, glucose metabolism	[20]
**PML**	Stress, cell growth, senescence	Affects apoptosis, cell growth, and senescence	Suppression	Cell growth, apoptosis, and senescence regulation	[21]
**MYC**	Cell growth, metabolic stress	Transcription al activity affects growth, metabolism	Activation	Cell growth, proliferation, metabolism regulation	[16]
**HMGB1**	DNA binding, stress	Affects gene expression regulation	Activation	DNA binding, gene regulation	[22]

**GATA (GATA3)**	Hematopoiesis, cardiac development	Regulates transcription of genes involved in hematopoiesis	Activation	Hematopoiesis, cardiac development	[23]
**MDM2**	p53 regulation, stress	Regulates p53 stability and activity	Suppression	p53 regulation, tumor suppression	[16]
**Nrf2**	Oxidative stress, detoxification	Enhanced antioxidant gene expression	Activation	Antioxidant response, detoxification	[24,25]
**TIP60**	DNA repair, transcription regulation	Affects chromatin structure and DNA repair response	Suppression	DNA repair, chromatin remodeling	[26]
**SREBPs**	Lipid metabolism, cholesterol synthesis	Regulates lipid homeostasis and cholesterol synthesis	Activation	Lipid homeostasis, cholesterol metabolism	[23]
**G6PD**	Pentose phosphate pathway, cellular redox balance	Regulates enzyme activity in the pentose phosphate pathway	Activation	Redox balance, cellular metabolism	[18]
**FOXM1**	Cell cycle, DNA damage	Affects transcriptional activity in cell cycle and DNA repair	Activation	Cell cycle regulation, DNA damage response	[27]
**CLOCK & BMAL1**	Circadian rhythm, gene expression	Affects circadian rhythm regulation	Activation	Circadian clock regulation	[28]

**SMADs**	TGF-β signaling, cell growth, differentiation	Regulates cell differentiation, apoptosis, growth	Suppression	Cell growth, differentiation, apoptosis	[29]
**ATF3/4**	Stress, DNA damage	Influences transcriptional activity related to stress and DNA damage	Activation	Stress response, DNA damage, apoptosis regulation	[30]

References:

1. Lakshminarasimhan, M.; Curth, U.; Moniot, S.; Mosalaganti, S.; Raunser, S.; Steegborn, C. Molecular architecture of the human protein deacetylase Sirt1 and its regulation by AROS and resveratrol. *Biosci. Rep.* **2013**, *33*, e00037. https://doi.org/10.1042/BSR20120121.
2. Hubbard, B.P.; Gomes, A.P.; Dai, H.; Li, J.; Case, A.W.; Considine, T.; Riera, T.V.; Lee, J.E.; E, S.Y.; Lamming, D.W.; et al. Evidence for a common mechanism of SIRT1 regulation by allosteric activators. *Science* **2013**, *339*, 1216–1219. https://doi.org/10.1126/science.1231097.
3. Sauve, A.A.; Wolberger, C.; Schramm, V.L.; Boeke, J.D. The biochemistry of sirtuins. *Annu. Rev. Biochem.* **2006**, *75*, 435–465. https://doi.org/10.1146/annurev.biochem.74.082803.133500.
4. Kang, H.; Suh, J.Y.; Jung, Y.S.; Jung, J.W.; Kim, M.K.; Chung, J.H. Peptide switch is essential for Sirt1 deacetylase activity. *Mol. Cell* **2011**, *44*, 203–213. https://doi.org/10.1016/j.molcel.2011.07.038.
5. Jasencakova, Z.; Meister, A.; Walter, J.; Turner, B.M.; Schubert, I. Histone H4 acetylation of euchromatin and heterochromatin is cell cycle dependent and correlated with replication rather than with transcription. *Plant Cell* **2000**, *12*, 2087–2100. https://doi.org/10.1105/tpc.12.11.2087.
6. González Cohens, F.; González Fuenzalida, F. The coronavirus pandemic did not impact Chilean organ donation system. *Transpl. Int. Off. J. Eur. Soc. Organ. Transplant.* **2021**, *34*, 1987–1988. https://doi.org/10.1111/tri.13999.
7. Rogakou, E.P.; Pilch, D.R.; Orr, A.H.; Ivanova, V.S.; Bonner, W.M. DNA Double-stranded Breaks Induce Histone H2AX Phosphorylation on Serine 139. *J. Biol. Chem*. **1998**, *273*, 5858–5868. https://doi.org/10.1074/jbc.273.10.5858.
8. Kuno, A.; Hosoda, R.; Tsukamoto, M.; Sato, T.; Sakuragi, H.; Ajima, N.; Saga, Y.; Tada, K.; Taniguchi, Y.; Iwahara, N.; et al. SIRT1 in the cardiomyocyte counteracts doxorubicin-induced cardiotoxicity via regulating histone H2AX. *Cardiovasc. Res.* **2023**, *118*, 3360–3373. https://doi.org/10.1093/cvr/cvac026.
9. Yamagata, K.; Kitabayashi, I. Sirt1 physically interacts with Tip60 and negatively regulates Tip60-mediated acetylation of H2AX. *Biochem. Biophys. Res. Commun*. **2009**, *390*, 1355–1360. https://doi.org/10.1016/j.bbrc.2009.10.156.
10. Oberdoerffer, P.; Michan, S.; McVay, M.; Mostoslavsky, R.; Vann, J.; Park, S.-K.; Hartlerode, A.; Stegmuller, J.; Hafner, A.; Loerch, P.; et al. SIRT1 redistribution on chromatin promotes genomic stability but alters gene expression during aging. *Cell* **2008**, *135*, 907–918. https://doi.org/10.1016/j.cell.2008.10.025.
11. Zhang, J.; Cao, Y.; Ren, R.; Sui, W.; Zhang, Y.; Zhang, M.; Zhang, C. Medium-Dose Formoterol Attenuated Abdominal Aortic Aneurysm Induced by EPO via β2AR/cAMP/SIRT1 Pathway. *Adv. Sci. Weinh. Baden-Wurtt. Ger.* **2024**, *11*, e2306232. https://doi.org/10.1002/advs.202306232.
12. Pazienza, V.; Borghesan, M.; Mazza, T.; Sheedfar, F.; Panebianco, C.; Williams, R.; Mazzoccoli, G.; Andriulli, A.; Nakanishi, T.; Vinciguerra, M. SIRT1-metabolite binding histone macroH2A1.1 protects hepatocytes against lipid accumulation. *Aging* **2014**, *6*, 35–47. https://doi.org/10.18632/aging.100632.
13. Boulard, M.; Storck, S.; Cong, R.; Pinto, R.; Delage, H.; Bouvet, P. Histone variant macroH2A1 deletion in mice causes female-specific steatosis. *Epigenetics Chromatin* **2010**, *3*, 8. https://doi.org/10.1186/1756-8935-3-8.
14. Chen, Y.; Zhao, W.; Yang, J.S.; Cheng, Z.; Luo, H.; Lu, Z.; Tan, M.; Gu, W.; Zhao, Y. Quantitative acetylome analysis reveals the roles of SIRT1 in regulating diverse substrates and cellular pathways. *Mol. Cell Proteom.* **2012**, *11*, 1048–1062. https://doi.org/10.1074/mcp.M112.019547.
15. Chen, H.; Ruiz, D.P.; Novikov, L.; Casill, A.D.; Park, J.W.; Gamble, M.J. MacroH2A1.1 and PARP-1 cooperate to regulate transcription by promoting CBP-mediated H2B acetylation. *Nat. Struct. Mol. Biol.* **2014**, *21*, 981–989. https://doi.org/10.1038/nsmb.2903.
16. Dong, S.Y.; Guo, Y.J.; Feng, Y.; Cui, X.X.; Kuo, S.H.; Liu, T.; Wu, Y.C. The epigenetic regulation of HIF-1α by SIRT1 in MPP(+) treated SH-SY5Y cells. *Biochem. Biophys. Res. Commun.* **2016**, *470*, 453–459. https://doi.org/10.1016/j.bbrc.2016.01.013.

17. Lan, F.; Cacicedo, J.M.; Ruderman, N.; Ido, Y. SIRT1 modulation of the acetylation status, cytosolic localization, and activity of LKB1. Possible role in AMP-activated protein kinase activation. *J. Biol. Chem.* **2008**, *283*, 27628–27635. https://doi.org/10.1074/jbc.M805711200.
18. Ghisays, F.; Brace, C.S.; Yackly, S.M.; Kwon, H.J.; Mills, K.F.; Kashentseva, E.; Dmitriev, I.P.; Curiel, D.T.; Imai, S.-I.; Ellenberger, T. The N-Terminal Domain of SIRT1 Is a Positive Regulator of Endogenous SIRT1-Dependent Deacetylation and Transcriptional Outputs. *Cell Rep.* **2015**, *10*, 1665–1673. https://doi.org/10.1016/j.celrep.2015.02.036.
19. Wang, Y.; Zhang, L.; Che, X.; Li, W.; Liu, Z.; Jiang, J. Roles of SIRT1/FoxO1/SREBP-1 in the development of progestin resistance in endometrial cancer. *Arch. Gynecol. Obstet.* **2018**, *298*, 961–969. https://doi.org/10.1007/s00404-018-4893-3.
20. Zhou, X.; Song, Y.; Zeng, C.; Zhang, H.; Lv, C.; Shi, M.; Qin, S. Molecular Mechanism Underlying the Regulatory Effect of Vine Tea on Metabolic Syndrome by Targeting Redox Balance and Gut Microbiota. *Front. Nutr.* **2022**, *9*, 802015. https://doi.org/10.3389/fnut.2022.802015.
21. Campagna, M.; Herranz, D.; Garcia, M.A.; Marcos-Villar, L.; González-Santamaría, J.; Gallego, P.; Gutierrez, S.; Collado, M.; Serrano, M.; Esteban, M.; et al. SIRT1 stabilizes PML promoting its sumoylation. *Cell Death Differ.* **2011**, *18*, 72–79. https://doi.org/10.1038/cdd.2010.77.
22. Wei, S.; Gao, Y.; Dai, X.; Fu, W.; Cai, S.; Fang, H.; Zeng, Z.; Chen, Z. SIRT1-mediated HMGB1 deacetylation suppresses sepsis-associated acute kidney injury. *Am. J. Physiol. Ren. Physiol.* **2019**, *316*, F20–F31. https://doi.org/10.1152/ajprenal.00119.2018.
23. Colley, T.; Mercado, N.; Kunori, Y.; Brightling, C.; Bhavsar, P.K.; Barnes, P.J.; Ito, K. Defective sirtuin-1 increases IL-4 expression through acetylation of GATA-3 in patients with severe asthma. *J. Allergy Clin. Immunol.* **2016**, *137*, 1595–1597.e7. https://doi.org/10.1016/j.jaci.2015.10.013.
24. Gerhart-Hines, Z.; Rodgers, J.T.; Bare, O.; Lerin, C.; Kim, S.-H.; Mostoslavsky, R.; Alt, F.W.; Wu, Z.; Puigserver, P. Metabolic control of muscle mitochondrial function and fatty acid oxidation through SIRT1/PGC-1alpha. *EMBO J.* **2007**, *26*, 1913–1923. https://doi.org/10.1038/sj.emboj.7601633.
25. Rodgers, J.T.; Lerin, C.; Haas, W.; Gygi, S.P.; Spiegelman, B.M.; Puigserver, P. Nutrient control of glucose homeostasis through a complex of PGC-1alpha and SIRT1. *Nature* **2005**, *434*, 113–118. https://doi.org/10.1038/nature03354.
26. Wang, J.; Chen, J. SIRT1 regulates autoacetylation and histone acetyltransferase activity of TIP60. *J. Biol. Chem.* **2010**, *285*, 11458–11464. https://doi.org/10.1074/jbc.M109.087585.
27. Lee, J.J.; Lee, H.J.; Son, B.H.; Kim, S.; Ahn, J.; Ahn, S.D.; Cho, E.Y.; Gong, G. Expression of FOXM1 and related proteins in breast cancer molecular subtypes. *Int. J. Exp. Pathol.* **2016**, *97*, 170–177. https://doi.org/10.1111/iep.12187.
28. Tong, X.; Zhang, D.; Arthurs, B.; Li, P.; Durudogan, L.; Gupta, N.; Yin, L. Palmitate Inhibits SIRT1-Dependent BMAL1/CLOCK Interaction and Disrupts Circadian Gene Oscillations in Hepatocytes. *PLoS ONE* **2015**, *10*, e0130047. https://doi.org/10.1371/journal.pone.0130047.
29. Xiong, S.; Cheng, J.C.; Klausen, C.; Zhao, J.; Leung, P.C.K. TGF-β1 stimulates migration of type II endometrial cancer cells by down-regulating PTEN via activation of SMAD and ERK1/2 signaling pathways. *Oncotarget* **2016**, *7*, 61262–61272. https://doi.org/10.18632/oncotarget.11311.
30. Li, Z.Y.; Liu, Y.; Wang, Y.Y.; Li, X.; Han, Z.N.; Hong, L.; Li, Y.S.; Cui, X. NOX4 stimulates ANF secretion via activation of the Sirt1/Nrf2/ATF3/4 axis in hypoxic beating rat atria. *Mol. Med. Rep.* **2022**, *25*, 84. https://doi.org/10.3892/mmr.2022.12600.
